# Unveiling the Structural and Optical Properties of MgAl_2_O_4_ Single Crystals Irradiated by Swift Heavy Ions

**DOI:** 10.3390/ma17020344

**Published:** 2024-01-10

**Authors:** Abdirash Akilbekov, Arseny Kiryakov, Alma Dauletbekova, Gulnara Aralbayeva, Aiman Akylbekova, Zhulduz Ospanova

**Affiliations:** 1 Department of Technical Physics, L.N. Gumilyov Eurasian National University, Astana 010008, Kazakhstan; agm_555@mail.ru (G.A.); aiman88_88@mail.ru (A.A.); zhulduz-ospan@mail.ru (Z.O.); 2 Ural Federal University, 21 Mira Str., Yekaterinburg 620002, Russia; arseny.kiriakov@urfu.ru

**Keywords:** swift heavy ions, MgAl_2_O_4_, optical properties, flare-up, photoluminescence

## Abstract

A synthetic single crystal of magnesium-aluminate spinel was irradiated perpendicularly to the (111) plane with swift heavy xenon ions with an energy of 220 MeV. The modified layer was attested based on Raman scattering spectra recorded while focusing on the surface. A decrease in surface crystallinity was observed, reflected in the changes in fundamental optical characteristics such as the band gap and the energies of static and dynamic disorder. In this study, we demonstrate, along with the modification of optical characteristics and the formation of a disordered layer, the creation of new optically active centers. The luminescent properties of these centers were analyzed. The effect of temperature flare-up in the 3.4 eV band of the excitation spectrum was determined. The low sensitivity of Cr^3+^ luminescence to SHI is demonstrated.

## 1. Introduction

The physicochemical properties of magnesium aluminate spinel MgAl_2_O_4_ (MAS) have been extensively studied, making it one of the most well-researched materials. MAS crystals are of great interest due to their exceptional optical properties, which result from their unique crystal lattice structure. Thus, MgAl_2_O_4_ forms a face-centered densely packed cubic lattice with an Fd-3m space group. The spinel structure consists of two cationic sublattices: one with magnesium ions (2^+^) in an oxygen tetrahedral environment and the other with aluminum ions (3^+^) in an octahedral environment [1]. Disorder of the crystal lattice caused by high-energy effects such as neutron irradiation, thermal annealing, and ion implantation results in changes to fundamental optical characteristics, including the width of the forbidden gap (E_g_) [2]. The bandgap energy (E_g_) of MAS is between 7.8 and 8.1 eV [3,4,5]. The indicated energies are in the vacuum ultraviolet range, which can complicate optical absorption interpretation. Therefore, researchers studying the optical characteristics of wide-gap crystals are often limited to the UV–visible–IR region.

In the case of thermal annealing or neutron irradiation, there is a contribution from high-energy impacts on a material throughout the investigated depth. Ion irradiation differs significantly in this aspect. Large ionic radii and the charge of accelerated ions lead to elastic collisions with ions of the crystal lattice. During collisions, the momentum of the accelerated ion reduces. Thus, the accelerated ions have a limited spatial effect on a material. By adjusting the technological parameters, it is possible to modify the near-surface layer in a controlled manner [6]. When heavy ions with fission fragment energies are used to irradiate a material, ion bombardment can simulate the interaction of the material with nuclear reaction products [7,8]. Therefore, the study of the interaction of swift heavy ions (SHIs) with radiation-resistant materials is an important research area in radiation physics.

In recent studies [9,10], it was demonstrated that when SHIs interact with MAS crystals, the near-surface layer undergoes a modification of approximately 10 μm. This modification leads to significant changes in the optical absorption spectra due to the formation of optically active centers and the induction of photoluminescence. It is believed that the formation of new photoluminescence centers results from a complex of oxygen vacancies with charge compensation due to electron capture (F_2_^+^). However, the behavior of the fundamental absorption edge under SHI exposure and the dynamics of its change with an increasing irradiation dose remain unclear.

The purpose of this study is to examine the vibrational and optical properties of single crystals of MAS (111) that have been irradiated by 220 MeV Xe ions. To achieve this, Raman spectra obtained from the surface layer were analyzed to verify and investigate the vibrational properties. The optical absorption edge behavior was also studied, and key parameters such as the band gap energy (E_g_) and Urbach energy (E_u_) were determined for crystals exposed to different fluences. Additionally, the photoluminescence characteristics were analyzed to understand their dynamics.

## 2. Materials and Methods

### 2.1. Sample Preparation and SHI Irradiation

Stoichiometrically pure single crystals of MgAl_2_O_4_ were provided by the German company “ALINEASON”. The dimensions of the samples are 10 × 10 × 0.5 mm, and the corresponding orientation is (111). The crystals contain impurities at a level of 50–100 ppm; such impurities can only be detected through impurity-sensitive measurement techniques such as photoluminescence spectroscopy. The samples were irradiated by 220 MeV Xe ions at room temperature and perpendicular to the (111) plane using cyclotron DC-60 (Astana, Kazakhstan). The fluence range was from 10^10^ to 10^13^ cm^−2^. In our previous study, SHIs were shown to modify structural characteristics to a depth of the order of 10 μm, with noticeable changes in the Raman vibrations dynamics [10]. This is in good agreement with SRIM modeling results.

### 2.2. Optical Investigation

#### 2.2.1. Raman Spectroscopy

Raman spectra (RS) were recorded using a LabRam HR800 Evolution confocal spectrometer (Horiba, Kyoto, Japan), and excitation was carried out with a 488 nm laser. Measurements were performed both at room temperature and at 83 K.

#### 2.2.2. Optical Absorption

Optical absorption spectroscopy in the VUV–UV–Vis range was conducted using a McPherson VuVAS 1000 PL spectrometer (Chelmsford, MA, USA) with a deuterium light source. The vacuum was maintained at no less than 10^–3^ Pa. Second-order diffraction from the grating monochromator was eliminated using a G-shaped filter with a boundary frequency of 220 nm (transmits light > 220 nm).

#### 2.2.3. Photoluminescence

The photoluminescence spectra were recorded using a Horiba Fluorolog 3 (Jobin Yvon, Edison, NJ, USA) spectrofluorometer equipped with a 450 W Xenon lamp, and typical signal-to-noise ratios greater than 20,000:1 were achieved. A Horiba Synapse CCD camera was installed as a registration detector. The sample was secured in a gold holder. The spectrometer was equipped with a special Janis optical cryostat with temperature expansion compensation. This enabled us to conduct PL measurements in the range from 4 K to room temperature.

## 3. Results and Discussion

### 3.1. Raman Spectra

The Raman light-scattering spectra obtained from the surfaces of both the unirradiated and irradiated samples show vibration modes that are typical of spinel with a cubic structure of $Fd\overset{ˉ}{3}m$. Table 1 summarizes the position and character of these oscillations. As the irradiation dose increases, the intensity of the vibrations decreases. When looking at the intensity-normalized spectra, a decrease in the signal-to-noise ratio can be observed. The unirradiated sample shows a clear signal with minimal noise, while the sample exposed to a fluence of 10^13^ cm^−2^ exhibits a high level of noise.

**Figure 1:**
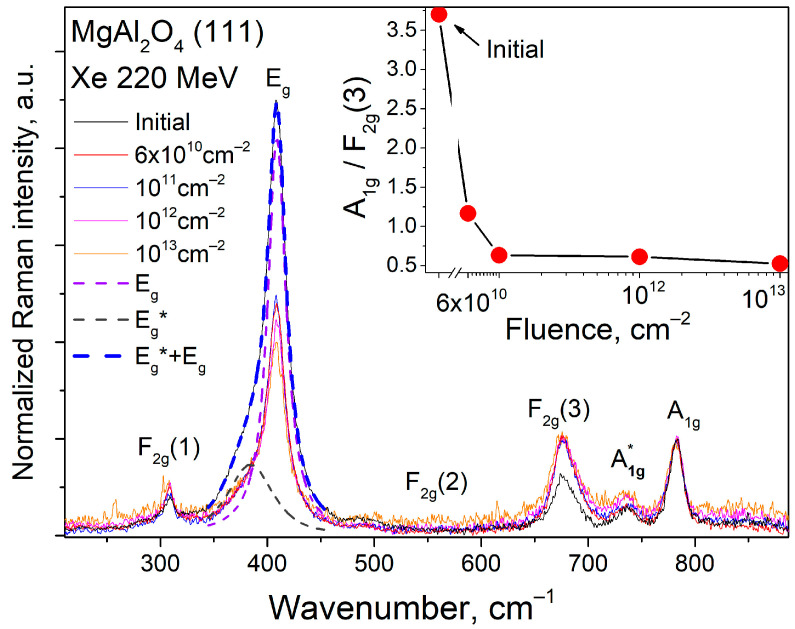
Raman light-scattering spectra of the original and SHI-irradiated MAS (111) crystals. Irradiation fluences are presented in colors. The decomposition of the main vibrational mode by the pseudo-Voigt function is indicated by dashed lines. The ratios of the areas of the A_1g_ to F_2g_(3) modes calculated from the spectra are given in the inset.

The Raman spectrum of the unirradiated crystal (Figure 1, black curve) shows that the basic mode E_g_ is broadened and has a full width at half maximum (FWHM_Eg_) of ~20 cm^−1^. In contrast, the value of this parameter for a natural crystal characterized by an extremely low content of intrinsic defects of the anti-site type FWHM_Eg_ is ~10 cm^−1^ [11] The main vibrational mode broadened, and an additional vibrational mode appeared as a shoulder at 386 cm^−1^ with an FWHM of approximately 50 cm^−1^. The simultaneous registration of A_1g_ vibrations suggests the presence of cationic mixing in the initial crystals. This mixing occurs due to the localization of the trivalent aluminum cation in the tetrahedral position of magnesium. The anti-site defects (ADs) ${Al}^{3+}{|}_{{Mg}^{2+}}$ and, vice versa, ${Mg}^{2+}{|}_{{Al}^{3+}}$ are formed. Defects of this type can be compensated for locally either by a nearby AD defect, if there is an excess of negative charge, or by an anionic vacancy, if there is a deficiency of positive charge. This is a characteristic phenomenon of artificially produced MAS. The crystals exhibited cation mixing defects due to high-temperature growth [12].

In our study, we utilized the occurrence of cationic mixing in the initial crystals to assess the Raman spectra in a qualitative manner. We normalized the intensity of the A_1g_ vibration mode, which is only slightly affected by cationic mixing, in the case of a secondary high-energy impact on spinel [12]. The A_1g_ mode occurs due to the symmetric stretching of the tetrahedral oxygen complex (breathing mode). The decreased sensitivity of oxygen tetrahedra to the formation of defects via cationic mixing is due to the peculiarities of the magnesium cationic sublattice. In spinel, magnesium cations form a diamond-like structure [13]. Several authors believe that the diamond-like magnesium cationic sublattice is responsible for the increased stability of MAS toward corpuscular impact [13,14]. In our studies, we assume that the mode associated with the tetrahedral cation, which has already undergone a high-energy change during crystal growth, remains unchanged after irradiation with SHI.

Figure 1 shows that as the SHI fluence increases, there is a decrease in the intensity of vibrational modes associated with defect-free MgAl_2_O_4_ and an increase in the intensity of vibrational modes due to induced cationic mixing in the spinel structure (A_1g_*). The authors of [12] observed a redistribution of intensities between the A_1g_ and F_2g_(3) modes. They interpreted the F_2g_(3) mode as a bending mode in the octahedron. The inset of Figure 1 shows that as the SHI fluence increases, the density of states of optical phonons causing the formation of F_2g_(3) increases, as indicated by the ratio of the areas of the modes. Apparently, the migration of some of the magnesium cations localized in oxygen tetrahedra to the void positions of oxygen octahedra occurred. The most significant changes in the presented relation occur up to a irradiation dose of 10^12^ cm^−2^.

**Table 1:** Raman modes in the original and irradiated MgAl_2_O_4_ crystals.

	F_2g_(1)	E_g_*	E_g_	F_2g_(2)	F_2g_(3)	A_1g_*	A_1g_
Position, cm^–1^	308		408	495	675	736	782
FWHM, cm^−1^	9.51	57	26	-	25	11	17
Possible motion	Transition of Mg atoms in the tetrahedral (T) sites ^a^	Motions connected with cation disorder ^a,b^	Asymmetric bending motion of the oxygen atoms within the T sites ^b^	Symmetric bending of the oxygens with respect to the cations in the T sites ^b^	Octahedral bending motion ^a^	Symmetric stretching (breathing) of the AlO_4_ tetrahedra due to cation disorder ^a^	Tetrahedral breathing mode ^a^

^a^—Ref. [15]; ^b^—Ref. [16].

Thus, Raman scattering spectroscopy focusing on the surfaces of the irradiated samples revealed a growth in cationic mixing because of the increasing SHI fluence. At the same time, at significant doses of the order of 10^12^ cm^−2^, a slowdown in the growth of cationic mixing was observed. There are competing processes for the formation of anti-site defects and their radiation annealing. Simultaneously, the decrease in the signal/noise ratio indicates a reduction in the crystallinity of the near-surface layer over the entire dose range investigated.

### 3.2. Optical Absorption

**Figure 2:**
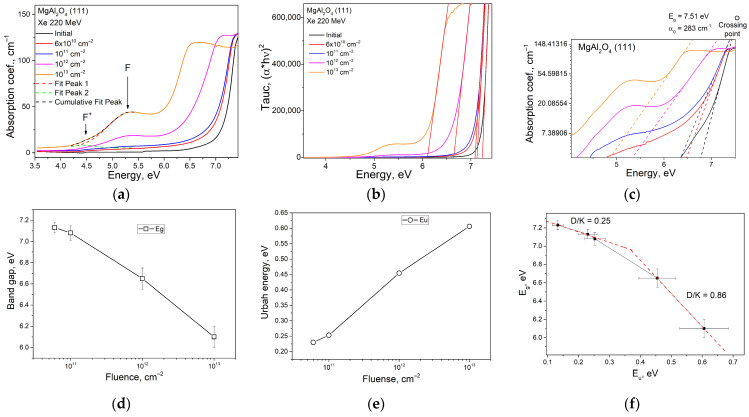
Optical absorption spectroscopy (**a**) and its derivatives. Optical absorption coefficient in Tauc coordinates (**b**) and in natural logarithm coordinates (Urbach coordinates) (**c**); variation in absorption edge (E_g_ (Fluence)) (**d**) and Urbach energy (E_u_ (Fluence)) (**e**) with increasing dose of SHI; E_g_ vs. E_u_ correlation curve (**f**).

The crystals under investigation were analyzed for their optical absorption, presented in the form of optical absorption coefficient spectra. This enabled a quantitative analysis of the data obtained as well as the calculation of fundamental material parameters such as the optical band gap and the average energy of static and dynamic disorder (Urbach energy). The spectral dependencies of the optical absorption coefficient of MgAl_2_O_4_ (111) crystals subjected to SHI are presented in Figure 2a. All the crystals were characterized by high transparency in the visible spectral range. However, significant changes were observed with an increasing irradiation dose in the investigated UV (3–6 eV) and VUV (6–9 eV) ranges. In the UV range, the formation of characteristic F-type centers, representing an anionic vacancy with one (F^+^) or two (F) trapped electrons, was observed. We studied the behavior of these types of centers in MgAl_2_O_4_ (111) crystals in detail in [10]. Particular attention should be paid to the VUV range, where the decrease in the energy of the fundamental absorption edge is clearly traced in Figure 2b. According to first-principles calculations, the extremum of the electron’s energy position in the valence band is located at the Gamma (Г) point of the first Brillouin zone. At the same value of the wave vector $\vec{k}$ (at the Г point), the minimum is situated in the conduction band [1,17]. The analysis of spectral dependences in Tauc coordinates, as shown in expression (1) [18], showed that direct interband transitions were realized (without a change in the wave vector $\vec{k}$), forming a clear linear section:

(1) $${\left(\alpha \cdot h\nu \right)}^{2}=A\cdot \left(h\nu -E_{g}\right)$$

where α is the absorption coefficient, hν is the photon energy, A is a constant, and E_g_ is the direct interband transitions (indicated by the multiplication index on the left-hand side).

Graphically, the variation in E_g_ with irradiation fluence is shown in Figure 2d. With an increasing irradiation dose, a linear trend of E_g_ decrease was observed, similar to the effects of temperature-induced changes in the optical gap. The blurring of the edge of the forbidden zone can occur due to two main factors—ion vibrations and lattice defects. In this case, the change in E_g_ depends on the displacement of atoms from their equilibrium position [19] and is described by expression (2):

(2) $$E_{0}\left(T,X\right)=K\left({\langle u^{2}\rangle }_{T}+{\langle u^{2}\rangle }_{X}\right)$$

where the coefficient K has the dimensionality of the second-order deformation potential constant, and $\langle u^{2}\rangle$ is the RMS displacement of atoms from the equilibrium position.

The experimentally observed results were interpreted within the framework of the Mott-FCO (Fritzsche–Cohen–Ovshinsky) model. The underlying principle of the model is that the valence band edges, as well as the conduction band edges, are formed by “tails” created by the disorder in the structure of the semiconducting material (i.e., a material characterized by a band gap) [20]. According to this model, atomic fluctuations, both temperature-induced and those caused by internal lattice imperfections, lead to a change in the nature of electronic states from extended to localized near the edges of the valence band and conduction band. These localized states appear as tails in the density of states, extending into the forbidden band [21]. Overall, it is assumed that optical transitions between electronic states in these tails are responsible for the Urbach tail region in absorption spectra [22].

The results in Figure 2c can be analyzed using the Urbach rule according to expression (3) [23,24]:

(3) $$\alpha \left(hv,T,X\right)=\alpha_{0}\cdot exp\left(\frac{hv-E_{g}\left(T\right)}{E_{U}\left(T,X\right)}\right),\;$$

where $\alpha_{0}$ is a constant, hv is the photon energy, $E_{g}\left(T\right)$ is the temperature-dependent function of the optical bandgap, and $E_{U}\left(T,X\right)$ is Urbach energy, characterizing the total static and dynamic disorder in the system.

The Urbach energy analysis conducted, as shown in Figure 2c, reveals two characteristic regions of dose dependence, which can be conditionally divided into “low fluences” and “high fluences”. For the “low fluences” region, there is a fan-shaped change in the linear section of the absorption coefficient at the scale of a natural logarithm, i.e., Urbach’s crystal rule [25], for which expression (4) is valid:

(4) $$\alpha \left(h\nu \right)=\alpha_{0}\cdot exp\left[\frac{h\nu –E_{g}}{K{\langle u^{2}\rangle }_{T}}\right]$$

where $\alpha_{0}$ and $E_{g}$ are the coordinates of the crossing point (the region of intersection of the extended sections of the linearized spectral range) determined from Figure 2c, hν is photon energy, and $K{\langle u^{2}\rangle }_{T}$ is Urbach energy mainly induced by the dynamical disorder (phonons) in the material. Table 2 presents the numerical values of constants obtained from the analysis of optical absorption spectra. The observed increase in Urbach energy at low fluences is due to the growth of the internal energy of the crystal, which was caused by the induction of disorder and the formation of new defects. However, when the fluences exceed 10^12^ cm^−2^, the crystalline Urbach rule is replaced by the glassy one [26]. This is clearly demonstrated by the parallel shift in linear regions of the optical absorption coefficient in natural logarithm coordinates. In this case, the disordered (amorphized) region of the crystal increases the fraction of static disorder. For the region corresponding to the “glassy” Urbach’s rule, expression (5) is valid:

(5) $$\alpha \left(h\nu \right)=\alpha_{g}\cdot exp\left[\frac{h\nu -E_{g}}{K{\langle u^{2}\rangle }_{X}}\right]$$

where $\alpha_{g}=\alpha_{0}\cdot exp\left[-\frac{E_{g}(0)}{E_{0}}\right]$, the temperature-independent logarithmic slope of the spectral response.

Figure 2e shows the linear relationship between Urbach energy and the applied fluence. The graph indicates that the Urbach energy increases linearly up to an irradiation fluence of 10^13^ cm^−2^. This suggests that there is a possibility of surface amorphization.

Upon analyzing expression (3), it is possible to express the value of the bandgap width through the constant of the second-order deformation potential (6) [24]:

(6) $$E_{g}\left(T,X\right)=E_{g}\left(0,0\right)-D\left[{\langle u^{2}\rangle }_{X}+{\langle U^{2}\rangle }_{T}\right],\;$$

where $E_{g}\left(0,0\right)$ denotes the bandgap magnitude in the absence of photon distortion. Considering expression (2), the equation (6) will be expressed in the form (7) [24]:

(7) $$E_{g}\left(T,X\right)=E_{g}\left(0,0\right)-\frac{D}{K}E_{U}\left(T,X\right),\;$$

Here, the coefficients D and K represent constants of the second-order deformation potential associated with the Urbach energy and the energy of optical transitions.

The correlation dependencies of the optical bandgap on the Urbach energy are presented in Figure 2f. It is evident that within the error range, for relatively low irradiation doses (up to 10^12^ cm^–2^), a linear correlation is observed, similar the dependence reported in [27]. The D/K ratio in this range is 0.25. However, at higher fluences, corresponding to the last two points on the correlation curve, the nature of the correlation dependency changes significantly, and in the range above a fluence of 10^12^ cm^–2^ D/K = 0.86 This suggests an increase in the contribution of structural disorder to the magnitude of E_g_ and implies that further increases in dose will more significantly alter the optical bandgap than Urbach energy.

By extrapolating the linearized range of the first three values on the correlation curve of Figure 2d to the intersection with the ordinate axis, one can estimate the value of E_g_(0,0), i.e., the energy of optical transitions without phonon distortions for the specific crystal. In this case, the value was determined to be 7.38 eV.

In our experiment, we observed a transition in the behavior of the tails of localized states from the crystalline Urbach rule to the glassy one as the irradiation fluence increased. The Urbach energy dependence in the glassy Urbach rule is usually determined by the temperature change in the classical view. However, when the ion irradiation dose is in the range of ≤10^12^ cm^−2^ in the spinel crystal, intrinsic defects form, which affect the growth of static disorder. This leads to a significant disorder of the crystal structure, as evidenced by the decrease in the intensity of vibrational modes in the Raman spectra (Figure 1). At fluences above 10^12^ cm^−2^, the optical absorption edge exhibits behavior typical of dynamic disorder in amorphous materials. This means that there is an increase in the dynamic disorder generated by the enhanced contribution of the phonon component.

### 3.3. Photoluminescence

**Figure 3:**
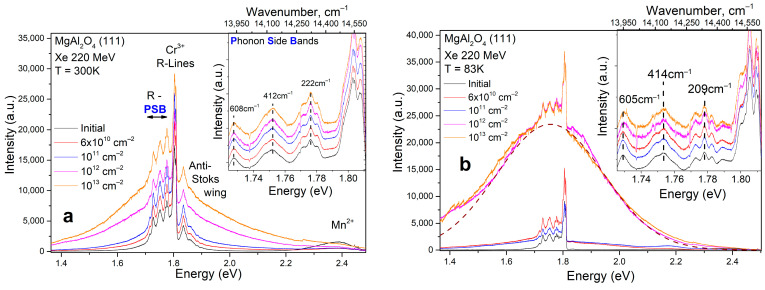
PL spectra upon laser excitation with energy Eex = 2.53 eV of MAS (111) crystals irradiated by 220 MeV Xe ions. The irradiation fluence is shown in color. Measurments made at room temperature (RT) are shown in (**a**), and those made at 83 K are shown in (**b**). The insets show the spectral regions corresponding to the Stokes wing of the phonon repetitions of the R-line of Cr^3+^.

The presence of optically active point defects can be conveniently detected using sensitive methods such as photoluminescence. Impurity point defects in the MgAl_2_O_4_ crystal, such as Cr^3+^ and Mn^2+^, were detected under intense laser excitation (E_ex_ = 2.53 eV) at room temperature and 83 K, as shown in Figure 3. These uncontrolled residual impurities exhibit intense PL signals due to high oscillator strength, lack of temperature, and concentration quenching. The initial sample shows a photoluminescent signal of Cr^3+^ ions, characterized by a phonon-free R-line. This R-line exhibits several variations due to different types of chromium ion arrangements in octahedral lattice nodes that experience trigonal distortions of octahedrons [28]. The Cr^3+^ ions in the spinel matrix have a D_3d_-type point symmetry. In the Stokes wing, phonon repetition bands have manifested due predominantly to acoustic phonons. The anti-Stokes wing shows less intense repetitions. When analyzing the main R-line and its variations, as well as phonon repetitions at room temperature, no changes in the position of the extrema and their spectroscopic features were found with increasing SHI fluence. Therefore, the chromium ions in the PL spectra at room temperature are excellent reference ions for qualitative comparative analyses.

In contrast, the intensity of the Mn^2+^ signal indicates an increased sensitivity to disorder [29]. It was observed that the signal of divalent manganese on the surfaces of the irradiated crystals decreased significantly with increasing fluence. This decrease was most likely due to the transfer of energy by impurity manganese ions to the newly formed defect states. A similar behavior of manganese centers is also known for ZnO crystals, where manganese centers are energy acceptors with the ability to transfer to Fe^3+^ ions [30,31]. In addition, manganese ions in spinel are characterized by a greater variation in the formation of polyvalent states (Mn^2+^, Mn^3+,^ and Mn^4+^) [32].

An interesting trend was observed: as the SHI fluence increased, an additional PL signal with a maximum at 1.7 eV was detected. At fluences of 10^12^ cm^−2^ and higher, a new optically active center formed, which was well detected using PL methods. A similar PL signal was also observed in a recent study [9]. The authors of [9] demonstrated that there is a distribution of new optically active centers according to the PL kinetics, which correspond to the nanosecond range.

In the low-temperature range, the PL signals exhibit the following behavior: the R-line of impurity ions Cr^3+^ remains constant, but the PL signal in the anti-Stokes wing of the Cr^3+^ R-line is completely quenched. Additionally, a slight shift in the position of the phonon repetition maximum at 605 cm^−1^ was observed in the Stokes region of the phonon wing. The most significant change was observed in the intensity of the new PL band, which, based on the spectral dependencies shown in Figure 3, is subject to temperature quenching. It is worth noting that increasing the implantation fluence by ten times does not affect the relative intensity of Cr^3+^ ions, but it does result in the formation of new types of centers. This indicates a significant change in the anionic sublattice. The reason for this change could be the disruption of crystallinity and the formation of an amorphous layer on the crystal surface.

As the fluence increases, the interaction dynamics between the crystal and accelerated ions undergo significant changes. This alteration is attributed to the heightened probability of the overlapping of the track regions of ions. Thus, considering the track of a single ion, it can be assumed that there is a normal distribution of the matrix-intrinsic defects from the track center to the periphery as a result of the knockout mechanism. An essential role in this case is played by secondary ionized atoms leading to defect cascades. Near the track center, the energy of secondary ionized atoms is high, inducing significant disorder (amorphization) of this region. As these secondary ionized atoms migrate from the track center towards its periphery, they induce the formation of point defects alone without disturbing the crystallinity. Therefore, at SHI fluences up to 10^12^ cm^−2^, the track density is insufficient to induce disorder in the spinel crystal lattice. However, with increasing irradiation fluence, the track density also increases, resulting in the appearance of overlap zones of tracks [33]. This leads to a sharp increase in amorphized parts. Consequently, there is a change in the mechanism of formation of Urbach’s tails of localized states from crystalline to glassy.

### 3.4. Excitation Spectra of PL

**Figure 4:**
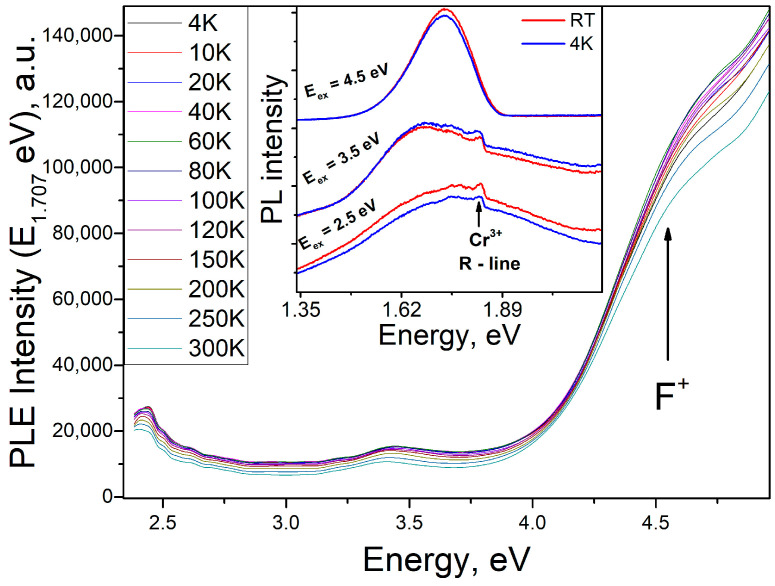
Excitation spectra of 1.707 eV PL band measured at different temperatures. The inset shows the PL spectra at RT (red) and 4 K (blue). The position of the chromium R-line is marked with an arrow.

The excitation spectra of broad band photoluminescence detected in the low-temperature PL measurements are shown in Figure 4.

**Figure 5:**
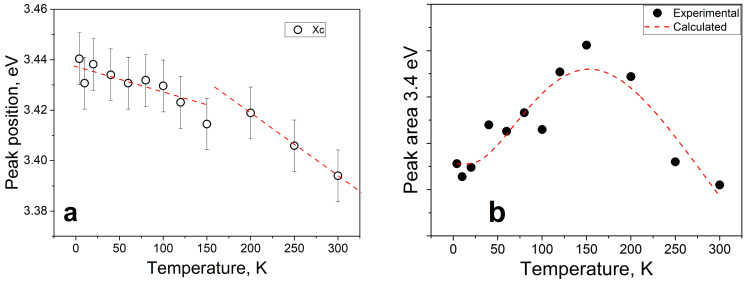
Temperature dynamics of the spectral characteristics of the 3.4 eV band in the PLE spectrum of MAS crystal irradiated with 220 MeV Xe ions. (**a**) shift of the peak maximum with increasing temperature; (**b**) change in the 3.4 eV peak area with increasing temperature.

The identified band is distinguished by several peaks, whose intensity and position exhibit variations with increasing temperature. Thus, an emission transition can be observed in the UV band, with excitation into the band of F^+^ centers. In addition, a selective band with a maximum energy of 3.4 eV was registered in the PLE (photoluminescence excitation) spectra, accompanied by a rise at energies exceeding 2.7 eV. The temperature changes in these bands are similar, suggesting their association with the same type of optically active center. We analyzed the temperature shift of the maximum (Figure 5a) and evaluated the area changes (Figure 5b) based on the example of the PLE spectral band at 3.4 eV. Beyond 2.7 eV, a notable increase in energy can also be observed. The temperature changes in these bands are similar, indicating that they belong to the same type of optically active center. We analyzed the temperature shift of the maximum (Figure 5a) and evaluated the area changes (Figure 5b) using the PLE spectral band at 3.4 eV as an example.

As the temperature increases, the 3.4 eV extremum moves towards the low-energy spectral region. Moreover, the rate of this shift increases between 200 and 300K. This suggests the presence of a defect with high electron–phonon coupling. An analysis of the PLE spectrum’s peak area in relation to temperature revealed a flare-up effect of photoluminescence as the temperature increases up to 150K. However, a further increase in temperature results in a decrease in the integral peak intensity of the 3.4 eV band. This phenomenon’s mechanism may be attributed to the presence of a two-level structure of the optically active center, where one level is predominantly radiative and the other is non-radiative. A similar dependence in the photoluminescence spectrum was observed by the authors of [34]. Later, this model was extended and explained by the authors of [35], where, in particular, an assumption about the physical meaning of pre-exponential multipliers was made. Using the methodological approach proposed by the authors of [34], the experimental results were approximated using Formula (8):

(8) $$I_{T}=I_{0}{\left\{\left[1+A*exp\left(\frac{E_{a}}{kT}\right)\right]\times \left[1+B*exp\left(-\frac{E_{nr}}{kT}\right)\right]\right\}}^{-1}$$

where A and B are multipliers characterizing the probabilities of level occupancy, E_a_ is the thermal activation energy (−0.0107 eV), and E_nr_ is the activation energy of the radiationless process (0.066 eV). The negative value of E_a_ indicates that the activation energy of the overlying level has smaller values than those for the underlying level Based on the analysis of the PLE spectra, we can conclude that the 1.7 eV broad PL band has a complex structure of energy levels. Apart from energy absorption via F^+^ centers, the optically active center is also characterized by its own energy levels, including a maximum energy of 3.4 eV, which is associated with an intra-center transition.

## 4. Conclusions

Synthetic single crystals of MgAl_2_O_4_ are characterized by cationic mixing, a phenomenon whose magnitude intensifies with increasing SHI fluence. Vacuum ultraviolet spectroscopy indicated the formation of intrinsic optically active centers as a result of irradiation with 220 MeV Xe ions. Notably, the optical gap decreased with an increasing irradiation dose as the internal energy of the crystal increased. It was found that at a fluence up to 10^12^ cm^−2^, the modification of optical characteristics was mainly due to the formation of intrinsic defects. Above this fluence, amorphization of the irradiated zone begins, which is apparently associated with the superposition of the track zones of SHI.

A recent study discovered the formation of a new optically active center in a modified layer. This center has a maximum at 1.7 eV, and its energy structure has undergone comprehensive analysis. The study found that there is an energy transfer from the excited F+ center to the new center. Additionally, an excitation spectrum registered a band at 3.4 eV, which directly refers to the excited state of the new center. This band is characterized by a flare-up to a temperature of 150 K, which may indicate that this center has a complex structure, including both radiative and non-radiative relaxation with competing processes between them.

## Figures and Tables

**Table 2:** Optical gap and Urbach energy for SHI-irradiated MAS crystals.

Fluence	Initial	6 × 10^10^ cm^–2^	10^11^ cm^–2^	10^12^ cm^–2^	10^13^ cm^–2^
E_g_, eV	7.23 ± 0.05	7.13 ± 0.05	7.08 ± 0.07	6.65 ± 0.1	6.10 ± 0.1
E_u_, eV	0.13 ± 0.017	0.23 ± 0.03	0.253 ± 0.032	0.454 ± 0.059	0.606 ± 0.078

## Data Availability

Data are contained within the article.
